# A high-throughput cloning system for reverse genetics in *Trypanosoma cruzi*

**DOI:** 10.1186/1471-2180-10-259

**Published:** 2010-10-13

**Authors:** Michel Batista, Fabricio K Marchini, Paola AF Celedon, Stenio P Fragoso, Christian M Probst, Henrique Preti, Luiz S Ozaki, Gregory A Buck, Samuel Goldenberg, Marco A Krieger

**Affiliations:** 1 Instituto Carlos Chagas, FIOCRUZ, Curitiba, Parana, Brazil; 2 Center for the Study of Biological Complexity, Virginia Commonwealth University, Richmond, Virginia, USA

## Abstract

**Background:**

The three trypanosomatids pathogenic to men, *Trypanosoma cruzi*, *Trypanosoma brucei *and *Leishmania major*, are etiological agents of Chagas disease, African sleeping sickness and cutaneous leishmaniasis, respectively. The complete sequencing of these trypanosomatid genomes represented a breakthrough in the understanding of these organisms. Genome sequencing is a step towards solving the parasite biology puzzle, as there are a high percentage of genes encoding proteins without functional annotation. Also, technical limitations in protein expression in heterologous systems reinforce the evident need for the development of a high-throughput reverse genetics platform. Ideally, such platform would lead to efficient cloning and compatibility with various approaches. Thus, we aimed to construct a highly efficient cloning platform compatible with plasmid vectors that are suitable for various approaches.

**Results:**

We constructed a platform with a flexible structure allowing the exchange of various elements, such as promoters, fusion tags, intergenic regions or resistance markers. This platform is based on Gateway^® ^technology, to ensure a fast and efficient cloning system. We obtained plasmid vectors carrying genes for fluorescent proteins (green, cyan or yellow), and sequences for the *c-myc *epitope, and tandem affinity purification or polyhistidine tags. The vectors were verified by successful subcellular localization of two previously characterized proteins (*Tc*Rab7 and PAR 2) and a putative centrin. For the tandem affinity purification tag, the purification of two protein complexes (ribosome and proteasome) was performed.

**Conclusions:**

We constructed plasmids with an efficient cloning system and suitable for use across various applications, such as protein localization and co-localization, protein partner identification and protein expression. This platform also allows vector customization, as the vectors were constructed to enable easy exchange of its elements. The development of this high-throughput platform is a step closer towards large-scale trypanosome applications and initiatives.

## Background

Currently, reverse genetics-based tools have been largely employed to obtain biological information on genes of unknown function. Nowadays genomic sequence data are easily obtained, but gene function is not always obviously extracted from these data. These tools have been used for many purposes, such as protein subcellular localization [1], protein interaction identification [2], protein overexpression [3], gene knockout [4] and gene silencing [5]. These techniques are particularly important in the study of trypanosomatid protozoa. Sexual reproduction, although not frequent, may play a role in the heterogeneity of several trypanosomatid species. However, these parasites mostly have a clonal population structure [6,7]. This characteristic precludes the use of forward genetics to study gene function in these parasites. In addition, their protein-coding genes are transcribed in polycistronic mRNAs, not related to bacterial operons, which are further processed to mature monocistronic mRNAs by a trans-splicing mechanism [8]. This process results in a short nucleotide sequence (miniexon) being added to the 5' end of trypanosomatid mRNAs [9]. The same machinery probably scans the intergenic region (IR) to process the upstream transcript and add the poly-A tail [10]. However, no consensus sequence for poly-A tail addition has been found in trypanosomes. Furthermore, gene expression in these microorganisms is mostly controlled by post-transcriptional events involving RNA processing and stability [8]. Hence, to be expressed in trypanosomatids, transgenes need to be flanked by intergenic regions that contain sequence elements promoting miniexon and poly-A tail addition.

Generally, IRs in trypanosomatid plasmid vectors are derived from constitutively expressed genes, such as those encoding glyceraldehyde 3-phosphate dehydrogenase [11,12], actin, aldolase [5,13,14], α-tubulin [15] or ubiquitin [16]. Gene expression in trypanosomatids appears to be ubiquitous and is not dependent on the presence of a typical RNA polymerase II (pol II) promoter [17]. Although typical pol II promoters have not been found in trypanosomatids, it has been shown that pol II transcription of an entire polycistronic unit initiates upstream of the first gene of the polycistron (in strand-switch regions) [18]. To enhance gene expression, vectors for use in trypanosomatids were constructed to ensure that transcription is directed by strong promoters like RNA polymerase I (pol I) promoters [3,14,19-21]. Some vectors were also designed to control gene expression, by combining T7 or pol I promoters with tetracycline-inducible systems [5,12,14,16,22-26]. These features require the development of reverse genetics strategies to deal with trypanosomatid biology.

There are a few examples of vectors designed for use in *T. cruzi*, mostly having conventional multiple cloning sites. Traditional molecular cloning methods, based on digestion by restriction enzymes and ligation by T4 DNA ligase, present various difficulties, such as low efficiency, limited number of sites for digestion and low adaptability for subcloning. Furthermore, other limitations have been observed in these plasmids, such as low flexibility to the exchange of elements like promoters, antibiotic resistance markers, fusion tags and IRs. These limitations become more evident during high-throughput procedures, where there is a need to adapt vectors, such that newly developed tags, alternate IRs and different resistance markers can be used. Taken together, these features reinforce the importance of producing reverse genetics tools, allowing quick and flexible strategies to better understand the biology of *T. cruzi*.

Recently, more efficient systems have been developed to circumvent some of the traditional cloning limitations. Two homologous recombination cloning systems, gap repair and the In-Fusion™ PCR Cloning Kit (Clontech, Mountain View, USA), have been used in high-throughput projects [27,28]. Other systems using site-specific recombination instead of homologous recombination, like the Creator™ DNA Cloning Kit (Clontech), Gateway^® ^technology (Invitrogen, Carlsbad, USA) and the Univector Plasmid-Fusion System [29], are other options. The use of cloning systems based on recombination instead of classic cloning techniques has improved the cloning process, making high-throughput projects less laborious.

The Creator and Univector cloning systems use Cre-*loxP *recombination [30], based on the recombination properties of bacteriophage P1. Gateway^® ^technology uses a distinct strategy, which is based on the recombinational properties of bacteriophage lambda [31]. Such site-specific recombination-based systems increase cloning efficiency and significantly decrease time spent on the work-bench. All site-specific recombination cloning systems present high cloning efficiencies, and the choice of system must take into account the features of each project.

Gateway(r) technology has been recently employed to create vectors for gene knockout [4] and protein subcellular localization [32] in *T. cruzi*. We developed a set of destination vectors employing Gateway(r) technology for use in reverse genetics. We validated our strategy using genes previously characterized in the literature through protein complex purification, and protein subcellular localization and co-localization techniques in *T. cruzi*.

## Results and Discussion

### Validation of vectors

We constructed a high throughput reverse genetics platform that can be easily modified for use in various trypanosomatid species. The platform represents a set of vectors based on Gateway(r) technology-associated site-specific recombination cloning. The expression vectors were initially prepared for use in *Trypanosoma cruzi*, due to particular characteristics of this parasite, such as RNAi absence. We used a general designation, p*Tc*GW, to describe the vectors; the specific designation of each vector was based on the tag and the resistance marker they carry (N for neomycin, and H for hygromycin B). Accordingly, the vectors p*Tc*GFPN, p*Tc*CFPN and p*Tc*YFPN, carry the tags for green, cyan and yellow fluorescent protein, respectively. The plasmids p*Tc*6HN, p*Tc*MYCN and p*Tc*TAPN carry the tags for hexahistidine, *c-myc *epitope and tandem affinity purification, respectively. All of these plasmids contain the gene encoding neomycin resistance (N). Correspondingly, p*Tc*GFPH carries the gene for GFP and for hygromycin B resistance. All constructs contained intergenic regions from the *T. cruzi *ubiquitin locus (*Tc*UIR) [33]. The choice of *Tc*UIR was based on: (i) its short size (278 bp); (ii) its use in another plasmid vector for *T. cruzi *[16]; and (iii) due to the participation of ubiquitin in many cellular processes, possibly during all the life cycle stages of *T. cruzi*, *Tc*UIR may enable the use of vectors in different life cycle stages of *T. cruzi *(although this was not addressed here). Vector constructs were verified using five *T. cruzi *genes, including those encoding the ribosomal protein L27 (*Tcr*L27), the α6 20S proteasome subunit (*Tc*pr29A), the paraflagellar component PAR 2, a putative centrin and the small GTPase Rab7 (*Tc*Rab7). The genes were inserted into p*Tc*GFPN, p*Tc*GFPH, p*Tc*CFPN, p*Tc*MYCN, p*Tc*6HN, and p*Tc*TAPN. The clones obtained were named TAPneo-*Tcr*L27 (*Tcr*L27 inserted into p*Tc*TAPN), TAPneo-*Tc*pr29A (*Tc*pr29A inserted into p*Tc*TAPN), GFPneo-PAR2 (PAR 2 inserted into p*Tc*GFPN), MYCneo-centrin (centrin inserted into p*Tc*MYCN), 6Hneo-centrin (centrin inserted into p*Tc*6HN), GFPhyg-PAR2 (PAR 2 inserted into p*Tc*GFPH), GFPneo-Rab7 (*Tc*Rab7 inserted into p*Tc*GFPN), and CFPneo-Rab7 (*Tc*Rab7 inserted into p*Tc*CFPN). As a control, we used p*Tc*GFPN and p*Tc*TAPN vectors, in which a previously inserted gene (a hypothetical protein - Tc00.1047053510877.30) was removed while preserving the *att*B recombination sites present in all clones. These controls were named GFPneo-CTRL and TAPneo-CTRL.

All constructs and clones obtained in this study were verified by DNA sequencing and no mutations were observed. The sequences were submitted to GenBank (the accession numbers are present in the methods section).

### DNA analysis of transfected *T. cruzi *cells

**Figure 1:**
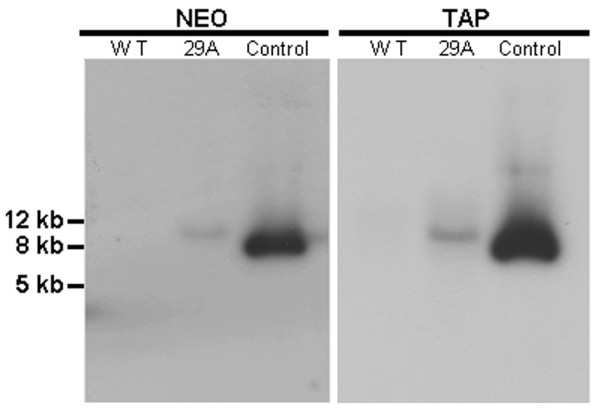
**Southern blot analysis of transfected *T. cruzi *cells**. Lanes represent *Hind*III-digested: genomic DNA from *T. cruzi *wild type (WT), from *T. cruzi *transfected with the TAPneo-*Tc*pr29A plasmid (29A) and TAPneo-*Tc*pr29A isolated plasmid (Control). The neomycin resistance marker (NEO) and the tandem affinity purification tag (TAP) were used as probes. 1 Kb Plus DNA Ladder (Invitrogen) was used as the molecular weight marker.

Southern blot assays were performed to analyze whether plasmid vectors were present as episomal or integrative forms after *T. cruzi *transfection. Genomic DNA from wild type *T. cruzi *and from cells transfected with TAPneo-*Tc*pr29A were digested with *Hin*dIII endonuclease, which rendered the linear plasmid. The neomycin resistance marker (NEO) and the tandem affinity purification tag (TAP) were amplified by PCR and used as probes to detect the presence of the vector. No band representing the linear plasmid (6.7 kb) was observed (Figure 1). Instead, the pattern obtained in Figure 1 shows the presence of one band, which is greater in size than the linear plasmid, suggesting that the regions represented by the probes were integrated into the *T. cruzi *genome.

This result was not surprising, as plasmid integration into the ribosomal locus has previously been shown in other constructs in which a ribosomal promoter was used [3,34]. Besides, there is also the possibility that the vectors were integrated into other areas of the *T. cruzi *genome, such as the ubiquitin locus, as the IRs (*Tc*UIR) for this locus were present in three copies in our constructs.

### Analysis of mRNA levels

**Figure 2:**
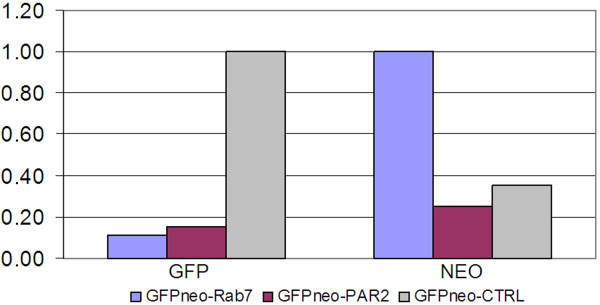
**Levels of GFP-fused and NEO recombinant mRNAs in *T. cruzi***. The Y-axis indicates the level of GFP and NEO mRNA quantified by real-time RT-PCR using populations of cells transfected with GFPneo-Rab7, GFPneo-PAR2 and GFPneo-CTRL.

To analyze mRNA levels for the GFP-fused recombinant protein in *T. cruzi *transfected with GFPneo-CTRL, GFPneo-Rab7 or GFPneo-PAR2, we performed real-time RT-PCR using oligonucleotides to amplify GFP. GFPneo-CTRL mRNA levels were approximately nine-fold higher than those of GFPneo-Rab7 and were six-fold higher than those of GFPneo-PAR2 (Figure 2). To better understand cell resistance without fluorescence, we quantified NEO mRNA levels in the same populations for which GFP mRNA levels were analyzed. Levels of NEO mRNA were greater than GFP mRNA in GFPneo-Rab7-transfected *T. cruzi *(Figure 2). Differences occurred despite all vectors containing a similar structure (i.e., IR sequences, resistance marker, protein tag and promoter). Also, although GFP-fused mRNAs are distinct, this is not the case for NEO mRNAs. This is an interesting point that still needs to be addressed.

### Detection of recombinant proteins and FACS analysis of transfected *T. cruzi*

**Figure 3:**
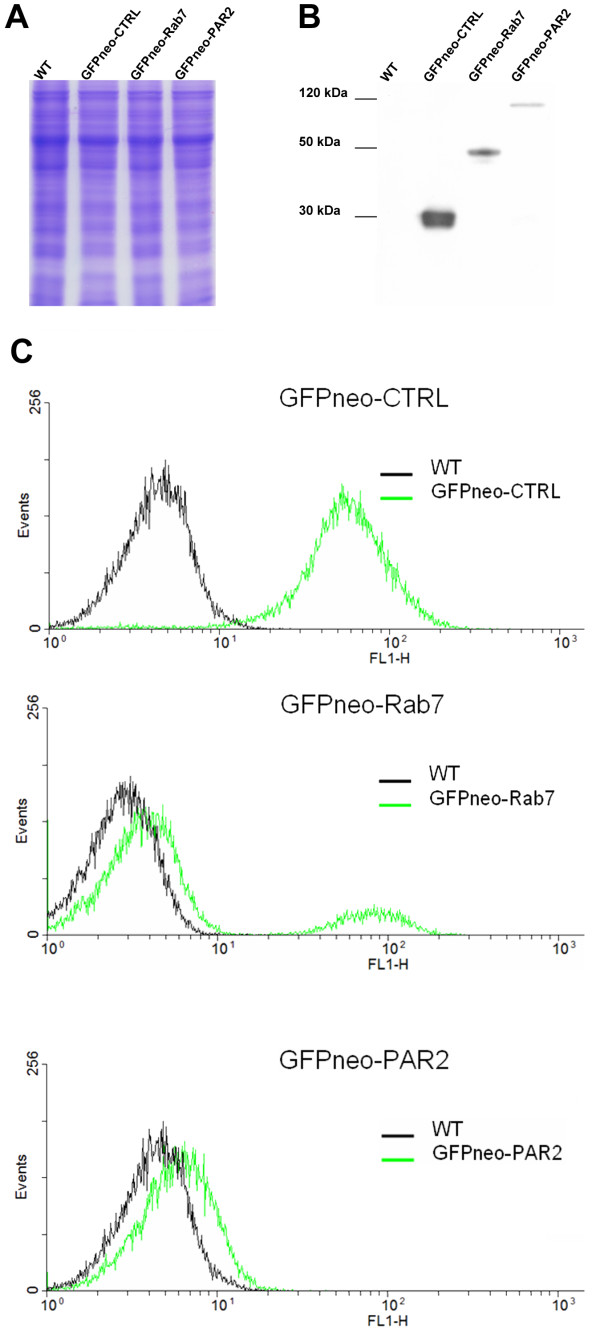
**Detection of GFP-fused recombinant proteins and FACS analysis**. Lanes in A and B represent protein extracts from *T. cruzi *wild type (WT) cells and cells transfected with GFPneo-CTRL, GFPneo-Rab7 and GFPneo-PAR2. In A is represented the load control gel. In B, these extracts were incubated with antibodies against GFP. BenchMark (Invitrogen) was used as the molecular weight marker. In C, *T. cruzi *wild type epimastigotes (WT) were used as a negative control. For each culture, 20,000 cells were counted. The Y- and X-axis represent the number of cells counted (events) and GFP fluorescence (FL1-H) in arbitrary fluorescence units (AFU), respectively.

To confirm the presence of recombinant proteins in transfected *T. cruzi*, western blot assays were performed using antibodies against the tags. The bands in Figure 3B correspond to the expected molecular weight of the PAR 2 and *Tc*Rab7 with addition of the GFP tag and the sequence for the *att*B1 site. Detection of *Tcr*L27 and *Tc*pr29A recombinant proteins (using anti-calmodulin binding peptide antibody) is shown in the "Tandem affinity purification" section, while the centrin recombinant protein used with *c-myc and *polyhistidine tags (using anti-*c-myc *and anti-histidine antibodies) are shown in Additional file 1 - Figure S1. Predicted molecular weight of native proteins *Tcr*L27, *Tc*pr29A, PAR 2, centrin and *Tc*Rab7, including the protein tags are described in Additional file 2 - Table S1.

*T. cruzi *transfected with GFP constructs were analyzed by cytometry, to verify the level of fluorescence in cells transfected with GFPneo-CTRL, GFPneo-Rab7 and GFPneo-PAR2 (Figure 3C). Cells transfected with GFPneo-CTRL had the highest percentage of fluorescent cells (96%), followed by GFPneo-Rab7 (19.7%) and GFPneo-PAR2 (2.6%). Fluorescence levels were correlated with protein intensity in western blots (Figure 3B).

To verify whether the amount of DNA used for transfection influenced the percentage of fluorescent cells, we analysed fluorescence in three cultures transfected with 15, 50 and 100 μg of the GFPneo-Rab7 clone. No fluorescence was detected by cytometry in any culture 48 h after transfection (data not shown). The fact that no fluorescence was detected in any of the transient assays may be explained by the integrative nature of our vectors. Episomal forms of an integrative vector are rapidly degraded after transfection [34]. However, after selecting for antibiotic-resistance in cells transfected with 15, 50 and 100 μg of the GFPneo-Rab7 plasmids, fluorescent cells were detected, but there was no correlation between the amount of DNA and fluorescence levels (data not shown). Thus, 15 μg of DNA appeared to be enough for transfections using the system described here.

### Subcellular localization of recombinant proteins

**Figure 4:**
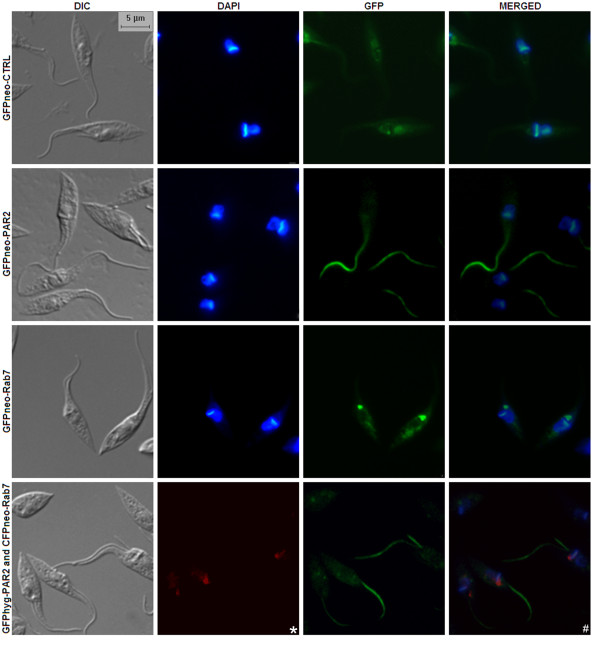
**Subcellular localization of *Tc*Rab7 and PAR 2 in *T. cruzi *using p*Tc*GW vectors**. Fluorescence microscopy of epimastigotes transfected with GFPneo-CTRL, GFPneo-PAR2, GFPneo-Rab7, GFPhyg-PAR2 and CFPneo-Rab7. The merged frame was composed by "GFP" and "DAPI" images overlap. The DAPI frame in the last row was replaced by a frame containing the cyan fluorescence-Rab7 construct (*), in which a red signal was used. The "#" frame contains a merger of DAPI/GFPhyg-PAR2/CFPneo-Rab7.

We selected genes whose subcellular localization is well known in epimastigotes. The small GTPase *Tc*Rab7 located in the anterior region of epimastigote cells at the Golgi cisternae, which appear in close proximity to the kinetoplast, basal bodies and flagellar pocket [35]. PAR 2 is a component of the *T. cruzi *paraflagellar rod located at the epimastigote flagellum [36]. We obtained identical localizations to those previously reported for both *Tc*Rab7 and PAR 2, using GFP and CFP fusions (Figure 4). GFPneo-CTRL was used as a control and showed a distribution pattern which was different from that for GFP-fused recombinant proteins. Although GFPneo-Rab7 was mostly located in the Golgi region, there was a signal in the cytoplasm, next to the nucleus. This may have been due to the overproduction of GFPneo-Rab7. *T. cruzi *transfected with both *Tc*Rab7 and PAR 2 in the same group of cells were also analyzed by fluorescence microscope. In this experiment, *Tc*Rab7 and PAR 2 were expressed from p*Tc*CFPN and p*Tc*GFPH, respectively. The results demonstrated the feasibility of protein co-localization in *T. cruzi *cells during a single transfection experiment using p*Tc*GW vectors (Figure 4). There was also no correlation between fluorescence intensity (Figure 4) and cytometry analysis data (Figure 3C). This absence of correlation was possibly caused by differences in exposure times and contrast (Figure 4). Indeed, we obtained the subcellular localization of a putative centrin of *T. cruzi *using the vector p*Tc*MYCN (Additional file 3 - Figure S2). This protein is related to centrosome and was located in epimastigotes near to kinetoplast in agreement with personal communication (Preti, H.).

Fluorescent proteins have been employed for subcellular localization in several types of organisms. This approach has some advantages: it is rapid and avoids the use of antibodies. However, in some cases, this technique may result in protein misallocation, due to at least two factors: (i) overexpression of recombinant proteins [37]; and (ii) interference of N- or C-terminal fusions with the localization signals [38,39]. To circumvent these problems, the platform described here was conceived for use with various strategies. First, recombinant vectors can be used without the pol I promoter, which may diminish expression of recombinant proteins.

Moreover, the IRs might be promoting different gene expression levels with the constructs in this study; thus, each IR could then be replaced by a non regulated or regulated IR, enabling standardized levels of expression or life cycle-specific expression, respectively.

Our group is currently employing deep sequence and proteomic analysis to select specific intergenic regions for use in p*Tc*GW vectors. Also, the analysis of gene sequences to detect particular localization signals may help to choose between N- or C-terminal fusions. The constructs in this study were designed for N-terminal fusions, but they can be modified quickly to generate C-terminal tags.

### Tandem affinity purification

**Figure 5:**
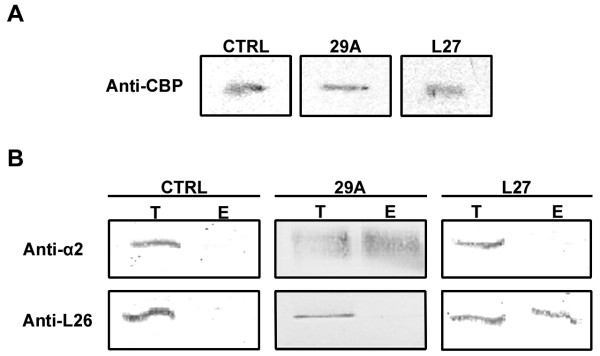
**Efficiency of L27 and 29A complexes purification with the original TAP tag tested in *T. cruzi *cells**. In A, the TAP tag-fused *Tcr*L27 (L27), *Tc*pr29A (29A) and the control TAPneo-CTRL (CTRL) was detected by western blot with anti-CBP antibody. In B, the fractions from TAP purification were probed with anti-L26 and anti-α2 in immunoblots. Lanes represent total protein (T) or eluted product after digestion (E). BenchMark (Invitrogen) was used as the molecular weight marker.

The tandem affinity purification (TAP) tag [40] comprises two repeated B domain of protein A (able to bind IgG), plus the site for TEV protease and the calmodulin binding peptide (CBP). The main reason for using a tandem purification approach is to avoid false positives. Two genes already described in the literature, *Tc*pr29A [41] and *Tcr*L27 [42] were inserted into p*Tc*TAPN. *Tc*rL27 encodes the L27 protein, a member of the larger ribosomal subunit, and *Tc*pr29A (29A) is a gene encoding the α6 20S proteasome subunit. The TAP tag-fused L27, 29A and the control TAPneo-CTRL (CTRL) were detected by western blot with anti-CBP antibody (Figure 5A).

A standard TAP procedure was followed to check the efficiency of both purification steps. The L27 resulting fractions were probed with anti-CBP antibody revealing an inefficient binding of the protein complex to the calmodulin column (second TAP step), as the TAP tag fused L27 protein was neither detected after the calmodulin column elution nor at the calmodulin beads (Additional file 4 - Figure S3). The low efficiency of protein recovery using CBP tag has been reported by other groups working with trypanosomatids [2].

Based on the partial success of the tags, all further tests were only performed up to the TEV digestion step (IgG column elution). The protein complex purification of *T. cruzi *transfected with TAPneo-*Tcr*L27, TAPneo-*Tc*pr29A and TAPneo-CTRL was performed using only the IgG column. To better evaluate this technique we used antibodies against other members of protein complexes probed. For the L27 ribosome enriched fraction we used antibody against L26 protein. The 29A proteasome-enriched fraction was probed with anti-α2 protein antibody. Antibodies against L26 and α2 were used in the same membrane for L27, 29A and CTRL complexes purification to make clear that the enrichment of the respective partners occurred just as a result of a protein-protein interaction and not as non-specific binding. L26 was only enriched during the L27 complex purification (Figure 5B). The same specificity was observed in the 29A purification, where α2 was exclusively detected (Figure 5B). Moreover, an absence of L26 and α2 during TAPneo-CTRL (vector expressing tags only) purification indicated that the newly expressed sequences were not generating nonspecific binding sites to L26 and α2 proteins (Figure 5B). Due to inefficiency of CBP tag column, we are currently testing other affinity tags, as a second step for tandem affinity purifications.

### General features of p*Tc*GW vectors

We constructed destination plasmid vectors with several N-terminal tags. The TAP, *c-myc*, polyhistidine, cyan and green fluorescent protein tags were successfully validated earlier in this study. These vectors have *attachment sites *for Gateway(r) recombination, providing several advantages over classic cloning, such as increases in speed and efficiency during the cloning step. Moreover, this platform allows ORF transference to destination vectors with distinct applications, providing different insights into protein function. The Gateway(r) platform has also had a significant impact on gene characterization in large-scale projects; for example: when a collection of ORFs has been available in compatible plasmids [37,43].

Another interesting feature was achieved during the design of vectors; we selected several one-cut restriction endonuclease sites to insert the elements, with the exception of *Xho*I whose sites flank the antibiotic resistance marker. This provides the flexibility to exchange all the elements in these vectors, such as promoter, intergenic regions (IRs), tags and antibiotic resistance genes. A good example of this flexibility was the set of experiments performed with the co-localization vector. This flexibility is important for further developments of this platform. Some of these developments have already been defined: First, there is evidence of intra-species ribosomal promoter specificity in *T. cruzi *[44]. Hence, we designed constructs allowing the exchange of the *T. cruzi *I ribosomal promoter with other promoters, such as the *T. cruzi *II ribosomal promoter, seeking to expand the use of p*Tc*GW vectors in other *T. cruzi *strains. Second, IRs are the other exchangeable elements in p*Tc*GW vectors. Several studies have shown that untranslated regions affect the level of expression of reporter genes in trypanosomatids [45-48]. The vectors described here allow IR exchange, thus modifying mRNA stability in attempts to modify the gene expression profiles in specific situations, for example during specific stages of the *T. cruzi *life cycle.

Finally, we followed a protocol for transfection that minimizes the amount of DNA and medium used. Thus, we obtained transfectants using DNA from a unique plasmid minipreparation. Moreover, our protocol also minimizes the amount of media and antibiotics used for cell cultivation, thus decreasing the cost and time-scale of large projects. Our procedure can be improved further, increasing its efficiency for use in high-throughput projects. Taken together, these observations demonstrate that our vector platform represents a powerful system for gene characterization in *T. cruzi*.

## Conclusions

Due to an absence of vectors combining a high-throughput cloning system and flexibility for exchanging its elements in *T. cruzi*, we developed and constructed destination vectors incorporating these features. Our p*Tc*GW vectors can be used for protein subcellular localization, co-localization and complex purification. These constructs can also be customized. In addition, we standardized some of our protocols, simplifying the use of our platform in large-scale projects. This is a very important step towards improving available methodologies for the characterization of thousands of genes whose functions remain unknown in *T. cruzi*.

## Methods

### Plasmid construction

**Figure 6:**
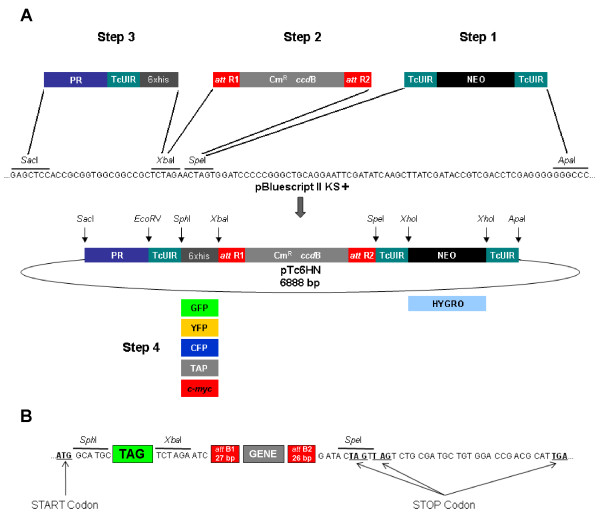
**Schematic drawing showing the vector construction steps**. The elements shown are the neomycin (NEO) and hygromycin (HYGRO) resistance genes, the *T. cruzi *intergenic region from ubiquitin locus (*Tc*UIR), the attachment sites for Gateway(r) recombination (*att*B1, *att*B2, *att*R1 and *att*R2), the chloramphenicol resistance gene (Cm^R^), the gene for negative selection during cloning (*ccd*B), the fusion tags (6xhis, GFP, YFP, CFP, TAP and *c-myc*) and the ribosomal promoter (PR). In A, the steps for vectors construction are represented. In B, the vector reading frame with start and stop codons are shown.

Three cassettes were inserted into the pBluescript(r) II plasmid (Stratagene, San Diego, USA) following the strategy shown in Figure 6. The cassette containing the neomycin resistance gene (NEO - 800 bp) flanked by a *T. cruzi *ubiquitin intergenic region (*Tc*UIR - 278 bp) and the cassette containing the *T. cruzi Dm*28c pol I promoter (617 bp) followed by a *Tc*UIR and a hexahistidine tag were synthesized *in vitro *(GenScript, Piscataway, USA) (Figure 6). The third DNA segment, represented by the RfA cassette (Invitrogen) (1711 bp), was PCR-amplified from pCR-Blunt and was inserted into pBluescript(r) II KS+. Restriction sites were placed in specific positions of the sequence, to insert the various cassettes or remove some segments of DNA, such that new segments could be inserted for the construction of new vectors.

The plasmid containing the three cassettes was named p*Tc*6HN. We constructed some derivative vectors from p*Tc*6HN, by replacing the polyhistidine tag with a TAP tag, the sequence of the *c-myc *epitope or with genes coding for fluorescent proteins (EGFP, CFP and YFP). All tags were amplified from plasmid vectors with the exception of *c-myc*, which was synthesized as two single-strand oligonucleotides (Additional file 5 - Table S2). For *c-myc *strands hybridization, 1.3 μg of each strand was used. The single strands were incubated in 10 mM NaCl buffer at 95°C for 10 min. The temperature was then slowly lowered to allow hybridization. After N-terminal tag insertion, the original vectors were identified as p*Tc*TAPN, p*Tc*GFPN, p*Tc*CFPN, p*Tc*YFPN, p*Tc*MYCN and p*Tc*GFPH (neomycin resistance was replaced with hygromycin resistance in p*Tc*GFPN). All of the constructs were sequenced by the commercial Macrogen facility (Macrogen, Seoul, Korea). The analysis of ab1 files was performed on SeqMan software (DNASTAR, Inc., Madison, USA). The sequences are available in GenBank under accession numbers HM162840 (p*Tc*YFPN), HM162841 (p*Tc*MYCN), HM162842 (p*Tc*TAPN), HM162843 (p*Tc*GFPN), HM162844 (p*Tc*GFPH), HM162845 (p*Tc*CFPN) and HM162846 (p*Tc*6HN). Oligonucleotides used for the construction and sequencing of vectors are listed in Additional file 5 - Table S2 and Additional file 6 - Table S3, respectively.

### Validation of vectors

Five *T. cruzi *genes were used in the validation process: *Tc*Rab7 (Tc00.1047053508461.270), PAR 2 (Tc00.1047053511215.119), a putative centrin (Tc00.1047053506559.380), *Tc*pr29A (Tc00.1047053506167.40), and *Tcr*L27 (Tc00.1047053506817.30). First, genes were amplified by PCR using oligonucleotides containing Gateway(r) *att*B sites (listed in Additional file 7 - Table S4). All genes had the stop codon inserted in the reverse oligonucleotide, with exception of centrin that uses the stop codon of vector. The PCR products were then inserted into pDONR 221 (Invitrogen) by BP recombination and then transferred to p*Tc*GW vectors by LR recombination. The *Tc*Rab7 gene was inserted into p*Tc*GFPN (for localization experiments) and p*Tc*CFPN (for co-localization experiments). The PAR 2 gene was inserted into p*Tc*GFPN (for localization experiments) and p*Tc*GFPH (for co-localization), while *Tc*pr29A and *Tcr*L27 were inserted into p*Tc*TAPN. The putative centrin was inserted into p*Tc*MYCN (for localization experiments), and into p*Tc*6HN. For construction of GFPneo-CTRL and TAPneo-CTRL, first, a hypothetical *T. cruzi *gene (Tc00.1047053510877.30) was inserted in these vectors. Then, this genetic element was removed by restriction endonuclease digestion (*Sma*I), preserving the *att*B recombination sites.

### Transfection of the parasites

Epimastigote forms of *T. cruzi Dm*28c were grown at 28°C in liver infusion tryptose (LIT) medium, supplemented with 10% fetal calf serum (FCS), to a density of approximately 3 × 10^7 ^cells ml^-1^. Parasites were then harvested by centrifugation at 4,000 × g for 5 min at room temperature, washed once in phosphate-buffered-saline (PBS) and resuspended in 0.4 ml of electroporation buffer pH 7.5 (140 mM NaCl, 25 mM HEPES, 0.74 mM Na_2_HPO_4_) to a density of 1 × 10^8 ^cells ml^-1^. Cells were then transferred to a 0.2 cm gap cuvette and 15 to 100 μg of DNA was added. For co-localization assays, 15 μg of each plasmid was used in the same cuvette. The mixture was placed on ice for 10 min and then subjected to 2 pulses of 450 V and 500 μF using the Gene Pulser II (Bio-Rad, Hercules, USA). After electroporation, cells were maintained on ice until being transferred into 4-10 ml of LIT medium containing 10% FCS, where they were incubated at 28°C. After 24 h of incubation, the antibiotic (hygromycin or G418) was added to an initial concentration of 125 μg ml^-1^. Then, 72 to 96 h after electroporation, cultures were diluted 1:10 and antibiotic concentrations were doubled. Stable resistant cells were obtained approximately 18 days after transfection.

### Southern blot analysis

DNA extraction was performed according to Medina-Acosta & Cross [49], with some modifications. Briefly, 1 × 10^8 ^cells were pelleted, washed once with PBS and lysed with 1.5 ml of TELT buffer (50 mM Tris-HCl, pH 8.0, 62.5 mM EDTA, pH 8.0, 2.5 M LiCl and 4% Triton X-100). DNA was purified three times using phenol/chloroform/isoamilic alcohol (v/v). After that, DNA was precipitated by adding 100% ethanol (1:1, v/v), then washed three times with 1 ml of 70% ethanol, dried at 25°C and resuspended in 100 μl of TE containing 10 μg ml^-1 ^RNase A.

*T. cruzi *DNA (10 μg) was restriction digested with *Hin*dIII (Amersham Biosciences, Piscataway, USA) and was resolved on a 0.8% agarose gel in TBE buffer. The DNA was transferred to nylon membranes (Amersham Biosciences) according to standard protocols [50]. Probes (NEO and TAP) were amplified (oligonucleotides listed in Additional file 8 - Table S5) and radioactively labeled with α-[P32]-dCTP (10 μCi/μl; 3,000 Ci/mmol) (Amersham Biosciences) using the Nick Translation System (Invitrogen), according to the manufacturer's instructions.

### Real-time RT-PCR

Total RNA was extracted from 1 × 10^8 ^cells by RNeasy Kit (Qiagen, Hilden, Germany) according to manufacturer's instructions. Single strand cDNA was obtained as follows: 1 μg of RNA and 1 μM oligo dT were mixed and incubated for 10 min at 70°C. Then, 4 μl of Improm-II buffer (Promega, Madison, USA), 3 mM MgCl_2_, 0.5 mM each dNTP, 40 U RNaseOUT (Invitrogen) and 2 μl Improm-II Reverse Transcriptase (Promega) were mixed in a final volume of 20 μl and incubated for 2 h at 42°C. The product was then purified with Microcon(r) YM-30 (Millipore, Massachusetts, USA) and resuspended with water at the concentration of 2 ng μl^-1^. PCR reactions included 10 ng or 0.4-50 ng (standard curve) of single strand cDNA samples as template, 0.25 μmol of each oligonucleotide, H2B histone oligonucleotides for normalization (listed in Additional file 8 - Table S5) and SYBR(r) Green PCR Master Mix (Applied Biosystems, Foster City, USA). A sample from *T. cruzi *wild type was used as a negative control. The reactions were performed and the standard curve was determined in triplicate and all PCR runs were carried out in an Applied Biosystems 7500 Real-Time PCR System. Data was acquired with the Real-Time PCR System Detection Software v1.4 (Applied Biosystems). Analysis was performed using an average of three quantifications for each sample.

### Western blot analysis

For immunoblotting analysis, cell lysates (from 5 × 10^6 ^parasites or, for TAP procedures, 5 to 15 μg of total protein and 25-50% of the digestion) were separated by SDS-PAGE using 13% polyacrylamide gels. Protein bands were transferred onto a nitrocellulose membrane (Hybond C, Amersham Biosciences) according to standard protocols [50]. Nonspecific binding sites were blocked by incubating the membrane for 1 h in 5% nonfat milk powder and 0.1% Tween-20 in TBS, pH 8.0. The membrane was then incubated for 1 h with either the monoclonal antibody anti-GFP (3.3 μg ml^-1^) (Molecular Probes(r) - Invitrogen), monoclonal anti-histidine (1.4 - 2.8 μg ml^-1^) (Amersham Biosciences), monoclonal anti-*c-myc *clone 9E10 (10 μg ml^-1^) (Clontech) or polyclonal serum anti-CBP (1:1,000) (Upstate(r)-Millipore) antibodies. For TAP procedures, polyclonal serum anti-L26 ribosomal protein [51] (1:250) and anti-α2 20S proteasome subunit (1:600) were used. The membrane was washed three times in TBS and was then incubated for 45 min with the secondary antibodies diluted in blocking solution. Secondary antibodies used included goat anti-mouse IgG conjugated with alkaline phosphatase (1:10,000) from Sigma, sheep anti-mouse IgG horseradish peroxidase-linked (1:7,500) or donkey anti-rabbit IgG horseradish peroxidase-linked (1:7,500) (GE Healthcare, Piscataway, USA). Bound antibodies were detected either with BCIP/NBT substrates for alkaline-phosphatase conjugated antibodies or the ECL Western blotting analysis system for horseadish peroxidase-linked antibodies (Amersham Biosciences), according to the manufacturer's instructions.

### Fluorescence Microscopy and FACS analysis of GFP expression

Epimastigote forms of transfected parasites were washed twice with PBS and resuspended to a final density of 5 × 10^7 ^cells ml^-1^. Cells were then added to the poly-L-lysine-coated cover slips, which were incubated at room temperature for 10 min. Cells were fixed with 4% paraformaldehyde for 15 min and in the last 5 min of this incubation, a solution of 2 μg ml^-1 ^DAPI, 0.1% triton X-100 was added to cells, which were then washed with PBS. For immunofluorescence assay, cells were processed as described up to the fixation. After this procedure, cells were incubated overnight with 25% goat serum diluted in PBS. Then, cells were incubated with monoclonal anti-*c-myc *antibody (40 μg ml^-1 ^in 25% goat serum diluted in PBS) (Clontech) for 1 h, washed three times with PBS and incubated with goat anti-mouse IgG antibody conjugated with Alexa Fluor(r) 488 (5 μg ml^-1^) (Invitrogen) for 1 h. After this, cells were incubated with 2 μg ml^-1 ^DAPI for 10 min and washed six times with PBS. Slides were mounted with 0.1% N-propyl-galacto and examined with a Nikon E600 microscope. For FACS analysis, epimastigote forms at growth log phase were counted on FacsCalibur (Becton Dickinson, San Jose, USA) until 20,000 events had been collected. Data was analyzed with WinMDI 2.9 (The Scripps Research Institute, San Diego, USA).

### TAP procedures

Total protein of epimastigote forms of *T. cruzi *cells transfected with TAPneo-*Tcr*L27, TAPneo-*Tc*pr29A and TAPneo-CTRL clones were used to check the efficiency of the TAP construct. For each culture, 4 × 10^9 ^cells were washed twice with ice-cold PBS and lysed at 4°C for 1 h with gentle agitation in lysis buffer (10 mM Tris-HCl, pH 8.0, 0.5 mM MgCl_2_, 50 mM NaCl, 0.5% NP-40, 10% glycerol, 0.5 mM DTT, 1 mM PMSF and 10 μM E64). All of the following steps were also carried out at 4°C. The lysate was centrifuged for 15 min at 10,800 × g to remove cell debris. The supernatant (total proteins) was transferred to a microcentrifuge tube (1.5 ml) and incubated with 50 μl of IgG Sepharose™ 6 Fast Flow bead suspension (GE Healthcare). After 2 h of ligation with gentle rotation, beads were washed three times with 1 ml of lysis buffer and once with the same volume of TEV buffer (50 mM Tris-HCl, pH 8.0, 0.5 mM EDTA, 1 mM DTT). Seventy units of AcTEV™ protease (Invitrogen) and 800 μl of TEV buffer were added to the beads and the tubes were left to rotate overnight to release the protein complex. Following digestion, the supernatant was transferred and the beads were washed two times with 200 μl of TEV buffer for maximum recovery. An aliquot of this digestion product (25%) was separated for western blot analysis.

The remaining digestion product was adjusted to a final concentration of 3 mM of CaCl_2 _and diluted with 3 volumes of calmodulin binding buffer (10 mM Tris-HCl, pH 8.0, 150 mM NaCl and 2 mM of CaCl_2_). The mix was incubated for 2 h at 4°C with 30 μl of a Calmodulin Sepharose™ 4B bead suspension (GE Healthcare). Following incubation, the flow through was saved and calmodulin beads were washed three times with 1 ml of calmodulin binding buffer. Proteins were eluted with calmodulin elution buffer (10 mM Tris-HCl, pH 8.0, 150 mM NaCl and 2 mM of EGTA) and the remaining beads were boiled with SDS-PAGE sample buffer. All fractions were TCA concentrated before analysis.

## Authors' contributions

MB and FKM participated in the design of the platform, the cloning process, the validation of vectors and drafted the manuscript. PAFC carried out the TAP procedures and helped to draft the manuscript. SPF participated in the cloning process and the Southern blot analysis and contributed to scientific discussion. CMP participated in the DNA sequencing analysis, the cloning process and contributed to scientific discussion. HP formatted the figures and contributed to vector validation. LSO, GAB and SG contributed to the design of the platform. MAK conceived the study, participated in the platform design and coordinated the project. All authors read and approved the final manuscript.

## Supplementary Material

Additional file 1 **Figure S1 - Detection of polyhistidine and *c-myc*-fused recombinant centrin**. Lanes represent protein extracts from *T. cruzi *wild type cells (WT), *T. cruzi *cells transfected with MYCneo-centrin and 6Hneo-centrin. These extracts were incubated with antibodies against (A) *c-myc *and (B) histidine. BenchMark (Invitrogen) was used as the molecular weight marker. Click here for file

Additional file 2 **Table S1 - Molecular weight of native and recombinant proteins**. Click here for file

Additional file 3 **Figure S2 - Subcellular localization of centrin using *c-myc *epitope tag**. Fluorescence microscopy of epimastigotes transfected with MYCneo-centrin. The merged frame was composed by "Anti-*c-myc*" and "DAPI" images overlap. Click here for file

Additional file 4 **Figure S3 - Tandem affinity purification efficiency**. Fractions of a complete L27 TAP purification were probed with anti-CBP antibody to follow the fusion protein and characterize the tags efficiency. 1 - wild type cells extract; 2 - transfected cells extract; 3 and 6 - flow through from IgG and Calmodulin columns, respectively; 4 and 7 - first and second washes from IgG and Calmodulin columns, respectively; 5 and 8 - third wash from IgG and Calmodulin columns, respectively; 9 - calmodulin beads; 10 - EGTA eluted. Fifteen micrograms of protein were loaded in lanes 1, 2 and 3; remaining fractions were TCA concentrated and 100% loaded. BenchMark (Invitrogen) was used as the molecular weight marker. Click here for file

Additional file 5 **Table S2 - Oligonucleotides for plasmid construction**. Click here for file

Additional file 6 **Table S3 - Oligonucleotides for sequencing of constructs and clones**. Click here for file

Additional file 7 **Table S4 - Oligonucleotides for Gateway(r) recombination**. Click here for file

Additional file 8 **Table S5 - Oligonucleotides for real-time RT-PCR and probe amplification (Southern blot)**. Click here for file
