# Supplementary figures and images for: A high-throughput cloning system for reverse genetics in Trypanosoma cruzi
Source: BMC Microbiol. 2010 Oct 13;10:259. doi: 10.1186/1471-2180-10-259 (PMC3020659; doi:10.1186/1471-2180-10-259)

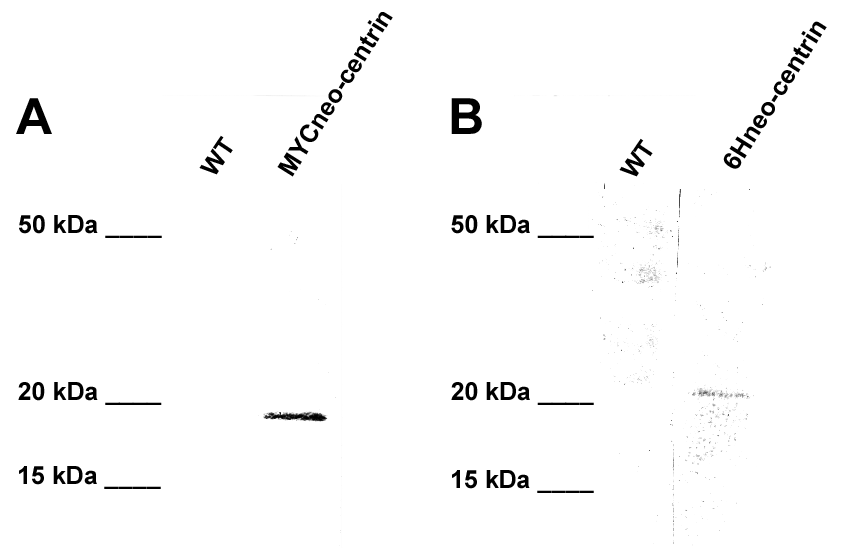

Supplement: Additional file 1 — Figure S1 - Detection of polyhistidine and c-myc-fused recombinant centrin. Lanes represent protein extracts from T. cruzi wild type cells (WT), T. cruzi cells transfected with MYCneo-centrin and 6Hneo-centrin. These extracts were incubated with antibodies against (A) c-myc and (B) histidine. BenchMark (Invitrogen) was used as the molecular weight marker. [file 1471-2180-10-259-S1.TIFF]

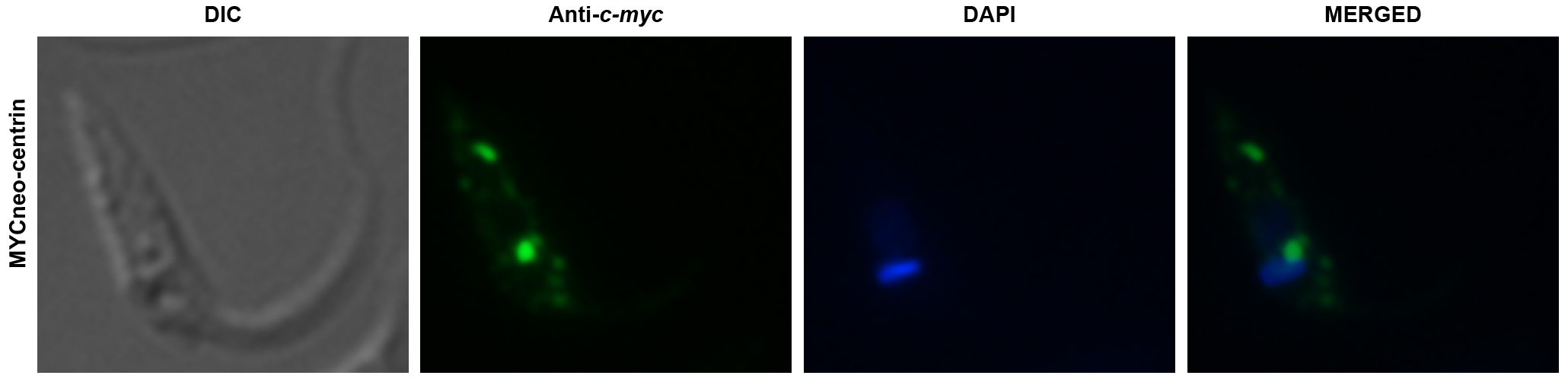

Supplement: Additional file 3 — Figure S2 - Subcellular localization of centrin using c-myc epitope tag. Fluorescence microscopy of epimastigotes transfected with MYCneo-centrin. The merged frame was composed by "Anti-c-myc" and "DAPI" images overlap. [file 1471-2180-10-259-S3.TIFF]

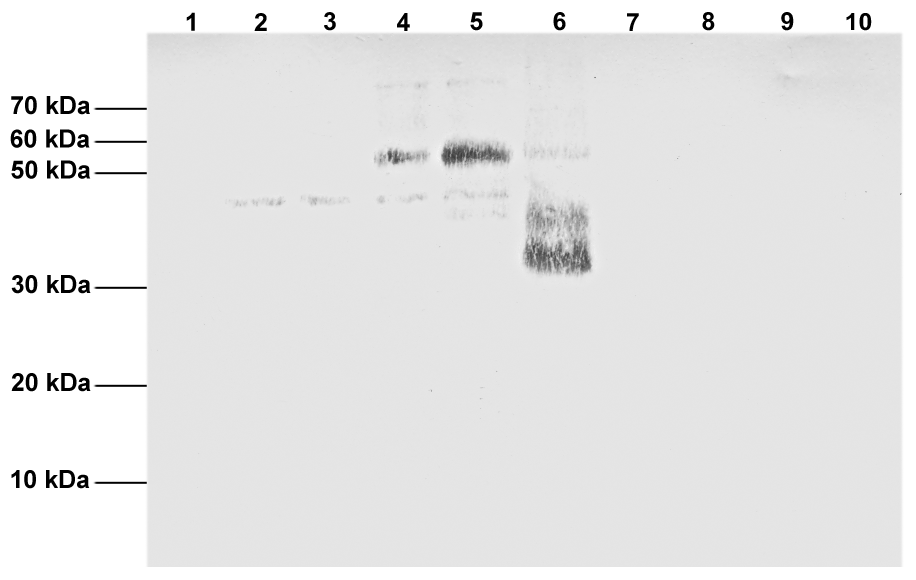

Supplement: Additional file 4 — Figure S3 - Tandem affinity purification efficiency. Fractions of a complete L27 TAP purification were probed with anti-CBP antibody to follow the fusion protein and characterize the tags efficiency. 1 - wild type cells extract; 2 - transfected cells extract; 3 and 6 - flow through from IgG and Calmodulin columns, respectively; 4 and 7 - first and second washes from IgG and Calmodulin columns, respectively; 5 and 8 - third wash from IgG and Calmodulin columns, respectively; 9 - calmodulin beads; 10 - EGTA eluted. Fifteen micrograms of protein were loaded in lanes 1, 2 and 3; remaining fractions were TCA concentrated and 100% loaded. BenchMark (Invitrogen) was used as the molecular weight marker. [file 1471-2180-10-259-S4.TIFF]
